# Impact of tobacco smoke constituents on nicotine-seeking behavior in adolescent and adult male rats

**DOI:** 10.3389/fpsyt.2023.1096213

**Published:** 2023-02-06

**Authors:** Candice A. Gellner, Diana Carreño, James D. Belluzzi, Frances M. Leslie

**Affiliations:** Department of Pharmaceutical Sciences, School of Pharmacy and Pharmaceutical Sciences, University of California, Irvine, Irvine, CA, United States

**Keywords:** adolescence, cigarette smoke, self-administration, reinstatement, drug-seeking

## Abstract

**Introduction:**

Given the rapid increase in teen vaping over recent years it is critical to understand mechanisms underlying addiction and relapse to tobacco use at this age. To evaluate the role of non-nicotine constituents in cigarette smoke, our lab has previously established a model of intravenous self-administration of aqueous cigarette smoke extract (CSE). We now compare the sensitivity of male adolescent and adult rats who have self-administered CSE or nicotine to reinstatement with the pharmacological stressor, yohimbine, with and without cues.

**Methods:**

Adolescents and adults, aged postnatal day (P) 34 and 84, were tested for the effect of yohimbine (0–2.5 mg/kg) on plasma corticosterone levels to establish a dose that was an effective stressor at both ages. Separate groups of animals were trained to lever press for food before beginning 1-hour drug self-administration sessions for nicotine or CSE (15 μg/kg/infusion nicotine content). Once stable responding was reached, drug was removed, and behavior extinguished. Drug-seeking behavior was reinstated with yohimbine, cues, or a combination of yohimbine and cues.

**Results:**

Although adolescents and adults showed different dose-responses for yohimbine-induced corticosterone release, a dose of 2.5 mg/kg increased stress hormone levels at both ages. Whereas both ages displayed similar responding for CSE and nicotine, adolescents self-administered more CSE and nicotine as compared to adults. Cues and cues + stress reinstated responding to a greater extent in animals that had self-administered CSE, regardless of age.

**Discussion:**

These findings suggest that non-nicotine tobacco smoke constituents influence later but not earlier stages of addiction in both adolescent and adult male rats.

## 1. Introduction

Tobacco addiction is a chronic relapsing disorder in which users continue to smoke despite negative health consequences (1, 2). Most smokers initiate tobacco use before the age of 18 (3, 4). Recent national surveys have demonstrated a large increase in the use of electronic cigarettes (e-cigarettes) among school age students and a concurrent decline in combustible cigarette use (5–7). There are several studies which have found a strong association between adolescents using e-cigarettes and subsequent cigarette smoking (8–12). It is well established that cessation of smoking is hard for teens, with 60–90% relapsing in the first year of cessation (13). However, teens who use e-cigarettes also report symptoms of dependence (14–18), which are highly correlated with salivary cotinine, a nicotine metabolite (18). Dependence is associated with lower odds of cessation of e-cigarette and conventional cigarette use (15, 49) demonstrating a need for a better understanding of mechanisms underlying adolescent relapse to tobacco and e-cigarette use.

To study relapse to smoking, animal models employ the use of triggers, such as stress, cues, or re-exposure to the drug. These triggers are known to cause relapse to smoking in humans and have also been shown to readily reinstate drug-seeking behavior in animals (19). Animal models of relapse to date have largely focused on reinstatement of drug-seeking behavior in adults, although clinical findings indicate that those who start smoking and try to quit as adolescents are more likely to relapse than adults (20, 21). Of the triggers that lead to relapse, stress has been identified as a major cause of relapse in humans (22). To study stress-induced relapse using animal models of tobacco dependence, researchers employ the use of foot-shock, a physiological stressor, or yohimbine, a pharmacological stressor, to induce stress. However, the α2 adrenergic receptor antagonist, yohimbine, has been shown to produce more reliable stress-induced reinstatement of drug-seeking behavior than foot-shock (23–25).

Our lab has shown that the presence of non-nicotine tobacco smoke constituents during self-administration enhances reinstatement induced by yohimbine + cues as compared to adult male rats that self-administered nicotine alone (26). However, the influence of non-nicotine tobacco smoke constituents on yohimbine-induced reinstatement of nicotine- or cigarette smoke extract (CSE)-seeking in adolescents is unknown. We have established a model of CSE self-administration in adolescent rats and have shown that the non-nicotine tobacco smoke constituents do not influence acquisition of self-administration at this age (27). We have now modified this experimental design to allow for assessment of reinstatement of drug-seeking behavior and have hypothesized that adolescents will show enhanced stress-induced reinstatement of nicotine- and CSE-seeking as compared to adults. As stress is an extremely influential trigger of relapse (28) it is vital to understand how stress influences both adolescents and adults, and whether prior exposure to non-nicotine tobacco smoke constituents influences this behavior.

## 2. Methods

### 2.1. Drugs

Nicotine hydrogen tartrate (Sigma, St Louis, MO) was dissolved in sterile saline and adjusted to pH 7.2–7.4. All nicotine doses were calculated as free base. CSE was created by bubbling smoke from commercial cigarettes (Camel unfiltered, RJ Reynolds) through sterile saline (26, 29). Briefly, eight cigarettes were smoked through 35 ml of saline solution (35-ml puffs over 2 s, repeated every 30 s) and the final solution was adjusted to pH 7.2–7.4. The CSE solution was prepared each day immediately before experimental testing in order to minimize differences resulting from differential stability of the constituents including nicotine (29). All CSE doses were defined by the solution’s nicotine content, which was analyzed by GC–MS (Finnigan Trace MS with Trace GC 2000 series, UCI Mass Spectrometry Facility). To ensure consistent nicotine concentration within CSE, periodic batches were sent to an outside facility (UCSF Clinical Pharmacology Laboratory) to confirm nicotine content. Yohimbine hydrochloride (Sigma-Aldrich) was dissolved in double distilled water.

### 2.2. Subjects

Male Sprague–Dawley rats were obtained from Charles River at postnatal (P) days 17 and 81. Adolescent rats remained with dam until weaning (P21). Upon weaning, animals were separated so that no more than one animal per litter per experimental group was used to avoid potential confounds. Animals were then group-housed throughout the experiment. All rats were maintained on a 12-h light/dark cycle (lights on at 07:00 am) with food and water available *ad libitum*. All experimental procedures followed NIH guidelines and were approved by the Institutional Animal Care and Use Committee of the University of California, Irvine.

Rats were minimally food-restricted beginning 2 days prior to operant conditioning to equalize the rat’s motivational states when they entered the daily experiments while still providing sufficient food to maintain their normal growth. Adolescent and adult rats were fed 15–25 or 20–25 g of food per animal per cage, respectively [based on Gellner et al. (27) and Mojica et al.(30)] to maintain normal growth during self-administration testing. Growth curves for both adolescents and adults followed normal trajectories (data not shown).

### 2.3. Dose–response of yohimbine-induced corticosterone release

Naive animals (P34 and 84, the age at which behaviorally tested animals were first exposed to nicotine or CSE) were handled for 3 days to minimize stress before being injected with yohimbine (0, 0.3, 0.8, or 2.5 mg/kg, i.p.). Sixty minutes later trunk blood was collected for corticosterone analysis using an ImmuChem Double Antibody Corticosterone ^125^I kit (MP Biomedicals, LLC). To minimize baseline corticosterone levels, testing took place within 2 h of lights on, when the 24-h rhythm of corticosterone levels are lowest (31).

### 2.4. Behavioral studies

#### 2.4.1. Apparatus

Animals were tested in plexiglass operant chambers (Med Associates, St Albans, VT), equipped with two levers. The required number of responses at the reinforced (R) lever turned on a cue light over the lever, turned off the house light, and activated an externally mounted syringe pump that infused drug. During the infusion (5.6 s yielding 100 μl of solution) and timeout period (20 s) the cue light remained illuminated, and the house light remained off. After the timeout period, the house light turned on, the cue light is turned off and signaled the availability of a reinforcer. Responses on the non-reinforced (NR) lever were recorded but had no consequences. Procedures modeled previous work done in our lab (26, 27).

#### 2.4.2. Food training

**Figure 1:**
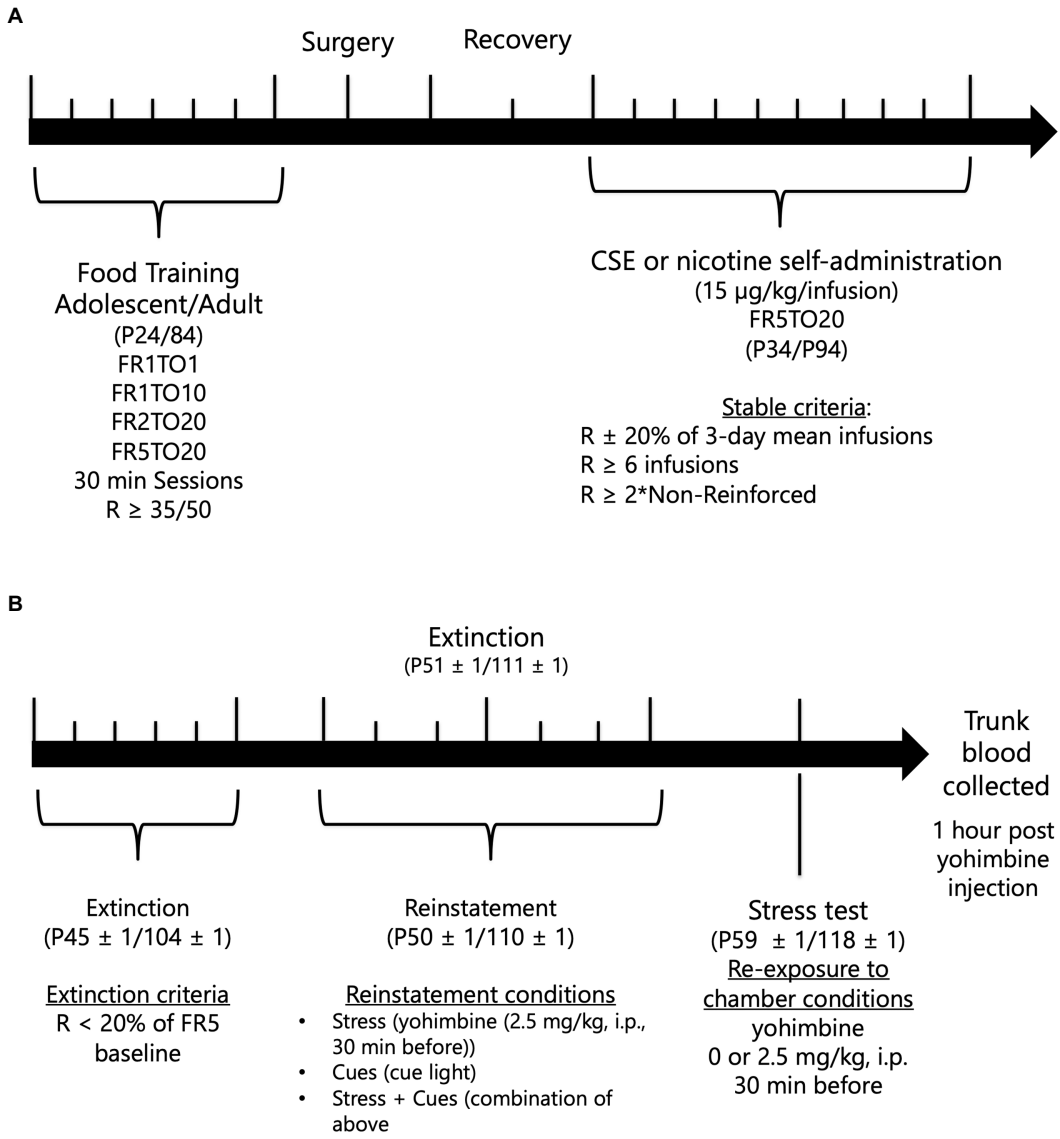
Timeline of behavioral experiment. A. Adolescent and adult rats underwent food training, catheter implantation, and self-administration of nicotine or CSE. B. After drug self-administration, extinction and stress-, cue-, and stress + cue-induced of nicotine- or CSE-seeking behavior before the final yohimbine exposure test occurred.

To facilitate learning of operant responding for drug-self-administration, adolescent and adult male rats, aged P24 and 84, respectively, were first trained to lever-press for food pellets (45-mg rodent purified diet; Bio-Serv, Frenchtown, NJ) under a fixed ratio 1 schedule with a 1-s timeout period (FR1TO1), followed by FR1TO10, FR2TO20, and completed with FR5TO20. Rats progressed to the next schedule when they earned at least 35 or 50 reinforcers (adolescents and adults, respectively) in two 30-min sessions per day. Timeline of experimental procedure is shown in Figure 1.

#### 2.4.3. Surgery

Following successful acquisition of food responding, rats were anesthetized with Equithesin (0.0035 ml/g body weight) and implanted with indwelling jugular vein catheters (32). After surgery, rats were given the analgesic carprofen (5 mg/kg, subcutaneous). During the 2-3-day recovery period, catheters were flushed daily with a heparinized saline solution to maintain patency. Catheter patency was tested for rapid (5–10 s) anesthesia by infusing propofol (5 mg/kg, i.v.) before and after the completion of self-administration experiments. Only animals showing rapid anesthesia were included in analyses.

#### 2.4.4. Drug self-administration, extinction and reinstatement of drug seeking

After recovery, adolescents and adults, aged P34 and 94, respectively, were allowed to self-administer nicotine or CSE [15 μg/kg/infusion nicotine content based on Costello et al. (26) and Gellner et al. (27)] at a FR5TO20 schedule during daily 1-h sessions. Rats self-administered nicotine or CSE for 10 days or until stable criteria were met (Number of infusions within 20% of the mean over 3 days, > = 2 × NR’infusions’, and > = 6 infusions). After reaching stable responding, extinction testing [house light on, animals not connected to the infusion tubing and responses on the levers had no consequence, based on Costello et al. (26)] began and lasted for 5 days or until extinction criteria were met (R < 20% of last FR5 responding). When adolescents and adults, aged P45 ± 1 and 104 ± 1, respectively, met extinction criteria, reinstatement testing began. Drug-seeking was reinstated using three conditions: stress only (2.5 mg/kg yohimbine, i.p. 30 min before session), cues only (reintroduction of cue light), or stress + cues [combination of the previous conditions based on Feltenstein et al. (33)] with a minimum of 2 days extinction between each test. Conditions were given in a within-subjects counterbalanced design.

Adolescents and adults, aged P59 ± 1 and P118 ± 1, respectively, who completed the reinstatement of drug seeking experiment then underwent a final test in which they were injected with yohimbine (0 or 2.5 mg/kg, i.p.), 30 min before being placed into the operant chamber with levers removed for 30 min before being decapitated. Trunk blood was collected to analyze corticosterone levels using an ImmuChem Double Antibody Corticosterone ^125^I kit (MP Biomedicals, LLC).

### 2.5. Statistical analyses

Yohimbine dose–response data were analyzed using a 3-way ANOVA on Corticosterone Level × Age × Dose. Significant main or interaction effects were further analyzed with a 2-way ANOVA for each Age separately on Corticosterone Level × Dose with Dunnett’s adjusted *post-hoc* comparisons. Self-administration at the FR5 schedule was analyzed using a 4-way ANOVA on R/NR Responses × Day × Age × Drug with repeated measures on R/NR Responses and Day. Significant main or interaction effects were further analyzed with a 2-way ANOVA for each Age or Day separately on R/NR Responses × Day or R/NR Responses × Age with Bonferroni-corrected paired (R/NR Responses and Day) or unpaired (Age) t-test *post-hoc* comparisons. Extinction data were analyzed using a 4-way ANOVA on R responses (as % of FR5 Responding) × Day × Age × Drug with repeated measures on Day. Significant main or interaction effects were further analyzed with a 2-way ANOVA for each Age or Day separately on R Responses × Day or R Responses × Age with Bonferroni-corrected paired (Day) or unpaired (Age) *t*-test *post-hoc* comparisons. Reinstatement data were analyzed using a 4-way ANOVA on R Responses (as % of FR5 Responding) × Condition × Age × Drug with repeated measures on Condition. Significant main or interaction effects were further analyzed with a 2-way ANOVA for each Drug or Condition separately on R Responses × Condition or R Responses × Drug with Bonferroni-corrected paired (Condition) or unpaired (Drug) *t*-test *post-hoc* comparisons.

## 3. Results

### 3.1. Yohimbine-induced corticosterone release

**Figure 2:**
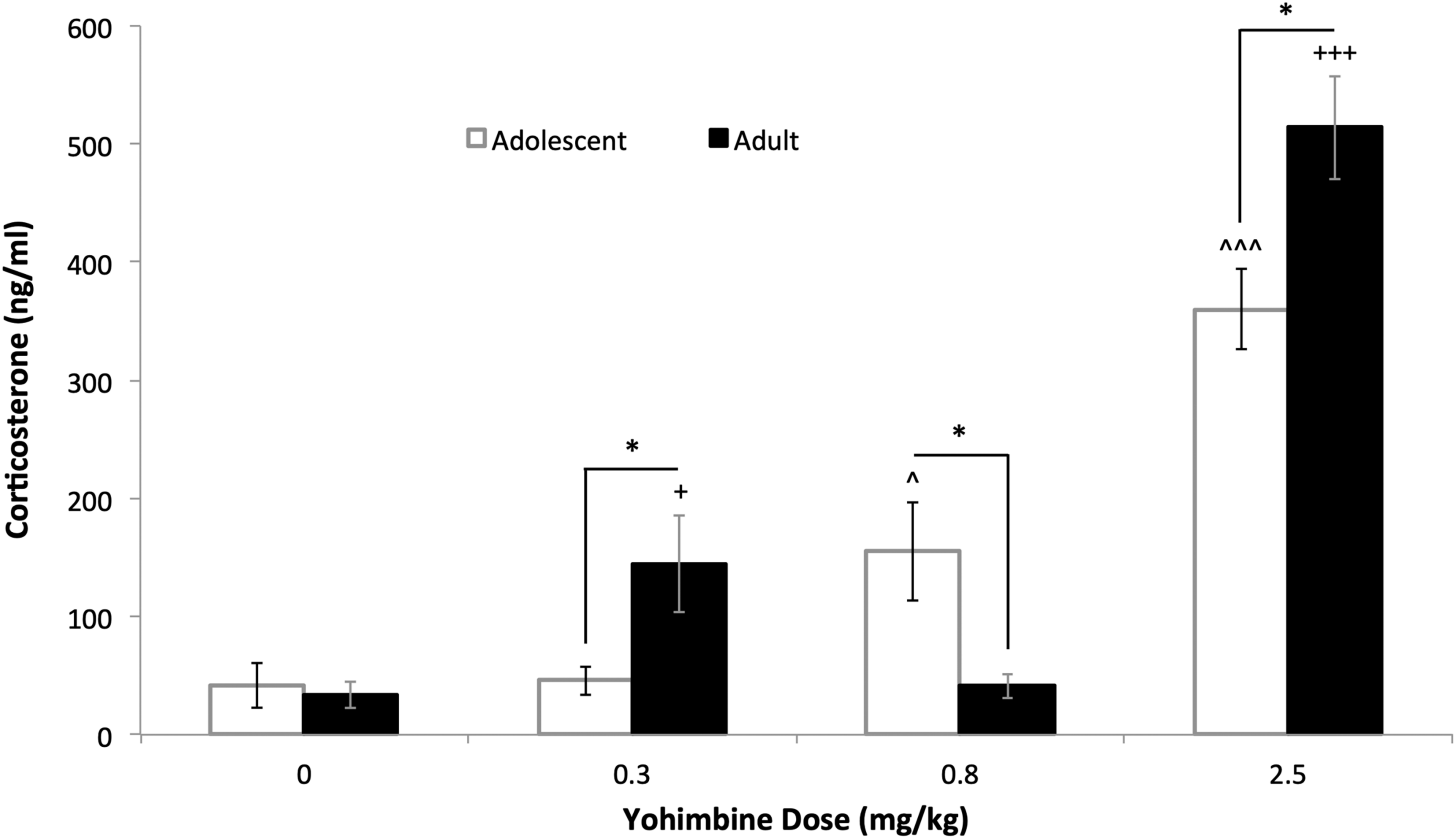
Age differences in yohimbine-induced plasma corticosterone secretion. The bars represent mean (±SEM) corticosterone levels after yohimbine injections for adolescent (white bars) and adult (black bars) rats. The α2 AR antagonist, yohimbine (0, 0.3, 0.8 and 2.5 mg/kg, i.p.) was given 1 h before trunk blood was collected and analyzed for corticosterone levels. ^ *p* < 0.05, ^^^ *p* < 0.0001 vs. 0 dose, adolescents; + *p* < 0.05, +++ *p* < 0.0001 vs. 0 dose, adults; * *p* < 0.05 vs. adult. *n* = 7–10 per group.

A dose–response analysis of yohimbine-induced corticosterone release was conducted in adolescent and adult male rats (Figure 2). Overall significant main effects of Dose [*F*(3,60) = 69.130, *p* < 0.0001] and an interaction between Age and Dose [F(3,60) = 7.239, *p* < 0.0001] were found. When each age was examined separately, both adolescents and adults displayed significant main effects of Dose [*F*(3,33) = 24.024, *p* < 0.0001 and *F*(3,27) = 51.178, *p* < 0.0001, respectively]. Basal corticosterone levels did not show an age difference. However, whereas adolescents showed a monotonic increase in yohimbine-induced corticosterone levels, adults showed a U-shaped function. In adolescents, yohimbine significantly increased plasma corticosterone levels at 0.8 and 2.5 mg/kg doses (*p* = 0.029 and *p* < 0.0001, respectively), whereas adults showed significant increases at 0.3 and 2.5 mg/kg doses (*p* = 0.048 and *p* < 0.0001, respectively). Plasma corticosterone levels in adolescents were significantly higher than adults following 0.8 mg/kg yohimbine (*p* = 0.024), but significantly lower at the 0.3 and 2.5 mg/kg doses (*p* = 0.048 and 0.013, respectively). Given that 2.5 mg/kg yohimbine induced significant corticosterone secretion in both adolescents and adults, this dose was chosen for use in subsequent experiments.

### 3.2. Initial self-administration

**Figure 3:**
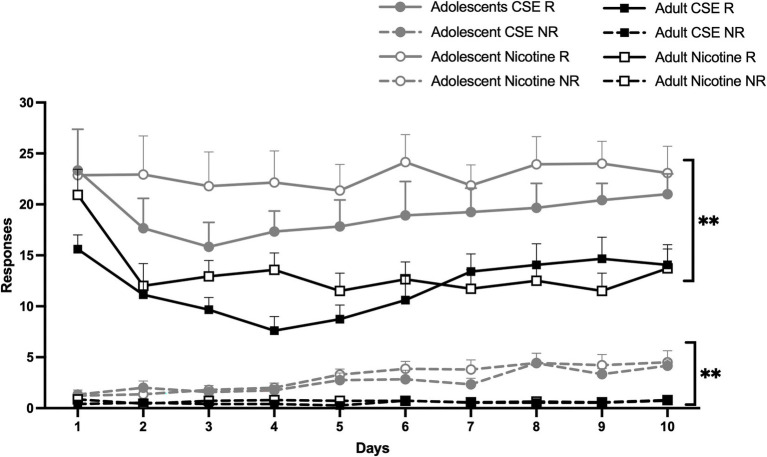
Age differences in the acquisition of self-administration. The lines represent mean (±SEM) responses leading to an infusion (R) of drug, nicotine or CSE, or not (NR) for adolescent (gray lines) and adult (black lines) rats. Open circles and open squares represent nicotine; closed circles and closed squares represent CSE. ** *p* < 0.005 vs. adult, on all days except Day 1. *n* = 12–15 per group.

Both adolescents and adults were found to achieve stable self-administration of nicotine and CSE over an initial 10-day period (Figure 3). Overall main effects of Days [*F*(9,459) = 7.118, *p* < 0.0001] and Age [*F*(1,51) = 25.332, *p* < 0.0001], with an interaction between Days × Age [F(9,459) = 4.326, *p* = 0.0001] were found. Since no overall or interaction effects of Drug were found, nicotine and CSE data were combined for the analysis, all groups are shown in Figure 3 for clarity. *Post-hoc* analysis showed that adolescents self-administered more drug and responded more on the NR lever compared to adults on all days except day 1 (*p* < 0.005; Figure 3). All animals reached stable self-administration of nicotine or CSE on Day 11 or Day 10, respectively; however only the first 10 days are shown (Figure 3).

### 3.3. Extinction and reinstatement

**Figure 4:**
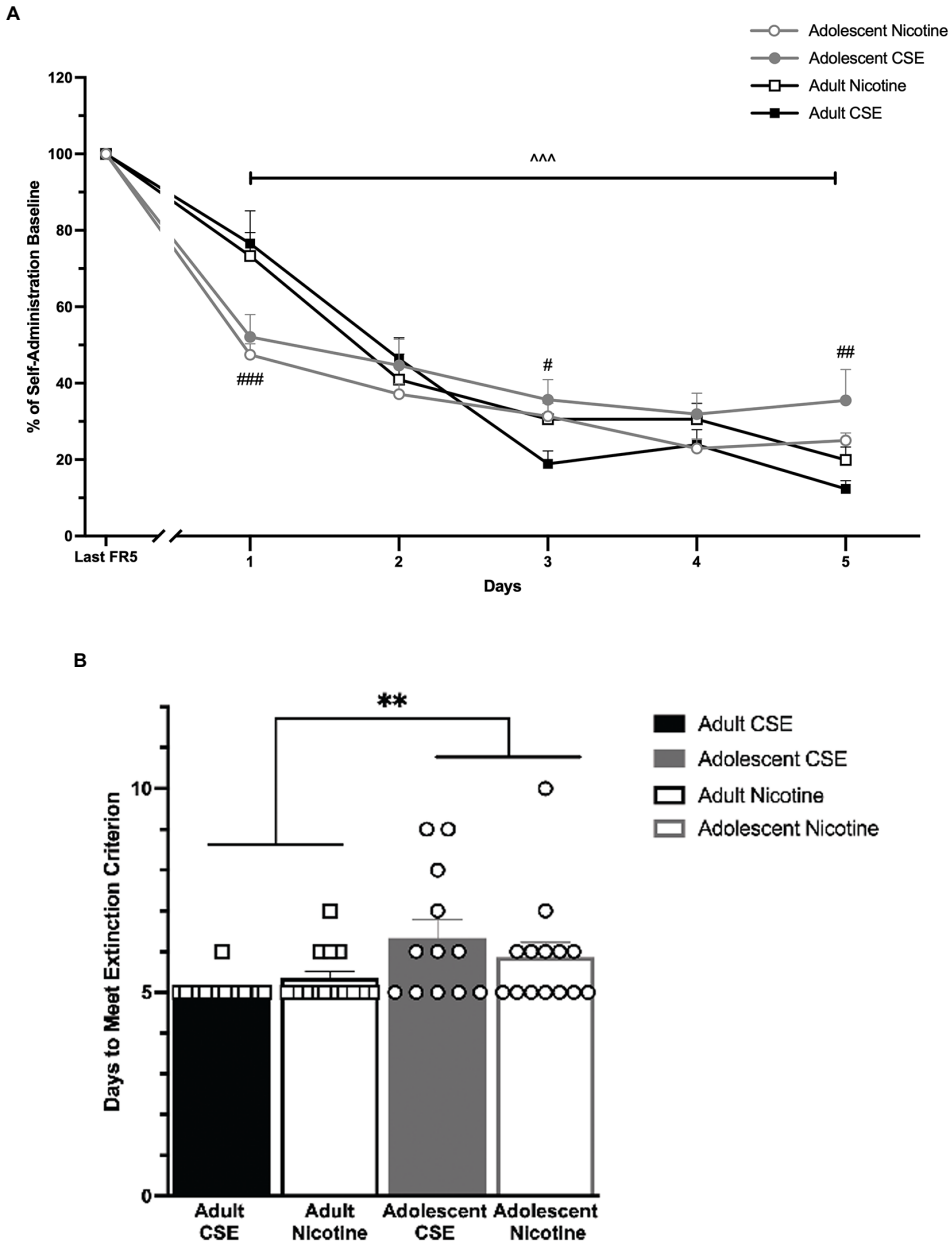
Age differences in extinction of responding. **(A)** The lines represent mean (±SEM) responding as a percentage of their last self-administration day for adolescent (gray lines) and adult (black lines) rats, which had previously self-administered nicotine or CSE. Drugs were collapsed. Open circles and open squares represent nicotine; closed circles and closed squares represent CSE. +++ *p* < 0.0001 vs. Last FR5. ### *p* < 0.0001, ## *p* < 0.01, # *p* < 0.05 vs. adult. *n* = 12–15 per group. **(B)** Days to meet extinction criterion. The bars represent mean (±SEM) days to meet extinction criterion in adolescents and adult of rats that self-administered CSE and nicotine. **p < 0.001 adults vs. adolescents. *n* = 12–15 per group.

Following stable self-administration, drug-seeking behavior was extinguished by removal of drug and cues (Figure 4). Data are reported as % of last day of FR5 responding since this baseline showed significant age differences. An overall main effect of Days [*F*(5,255) = 244.298, *p* < 0.0001] and an interaction between Days × Age [F(5,255) = 11.845, *p* < 0.0001] were found. Since no significant overall or interaction effects of Drug were found, nicotine and CSE data were combined for analysis, showing all groups for clarity (Figure 4A). Post-hoc analysis showed that all animals significantly reduced their responding on the reinforced lever starting on Day 1 and continuing through the end of extinction (*p* < 0.0001). However, adolescents extinguished significantly faster than adults on Day 1 (*p* < 0.0001), but less than adults on Days 3 and 5 (*p* = 0.037 and *p* = 0.002, respectively). Drug-seeking behavior was fully extinguished on average by Day 6 or Day 5 by adolescent or adult animals, respectively; however only the first 5 days are shown (Figure 4B).

**Figure 5:**
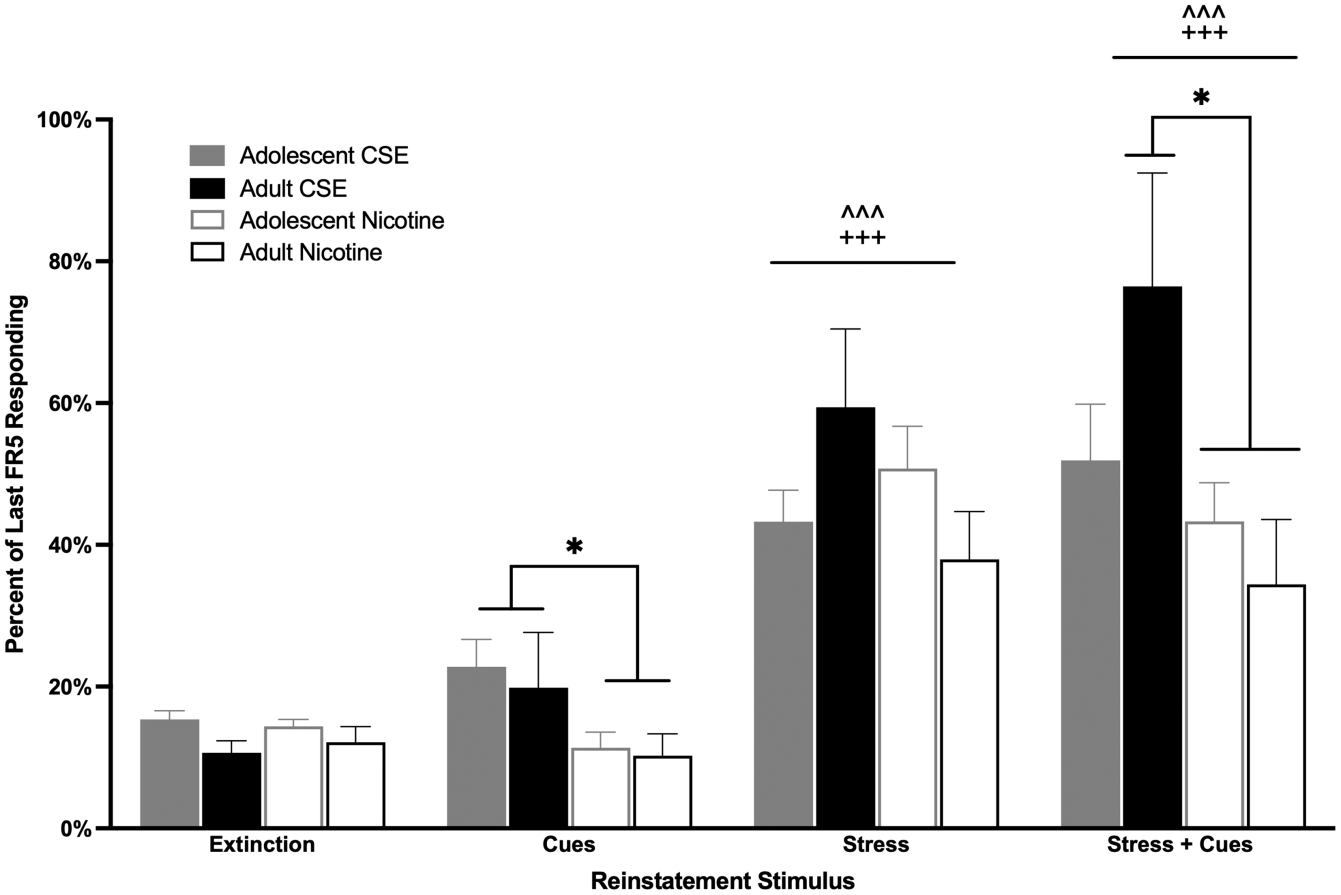
Drug differences in cue- and stress + cue-induced reinstatement. The bars represent mean (±SEM) responding on previously reinforced levers, adolescents and adults, that had self-administered CSE (closed bars) or nicotine (open bars). ^^^ *p* < 0.0001 vs. extinction, +++ *p* < 0.0001 vs. cue; * *p* < 0.05 CSE vs. nicotine. *n* = 12–15 per group.

Following extinction, animals were triggered to reinstate drug-seeking behavior with yohimbine stress, cues, or the combination of stress and cues (Figure 5). Overall main effects of Condition [*F*(3,153) = 37.399, *p* < 0.0001] and Drug [*F*(1,51) = 6.437, *p* = 0.014] were found. Since no overall or interaction effects of Age were found, adolescent and adult data were combined for analysis (Figure 5). Both stress and the combination of stress and cues induced reinstatement of both CSE- and nicotine-seeking behavior (*p* < 0.0001). Animals that had self-administered CSE responded more for cues and the combination of stress + cues compared to those that had worked for nicotine (*p* = 0.044 and *p* = 0.020, respectively; Figure 5).

### 3.4. HPA axis activation after stress-induced reinstatement

**Figure 6:**
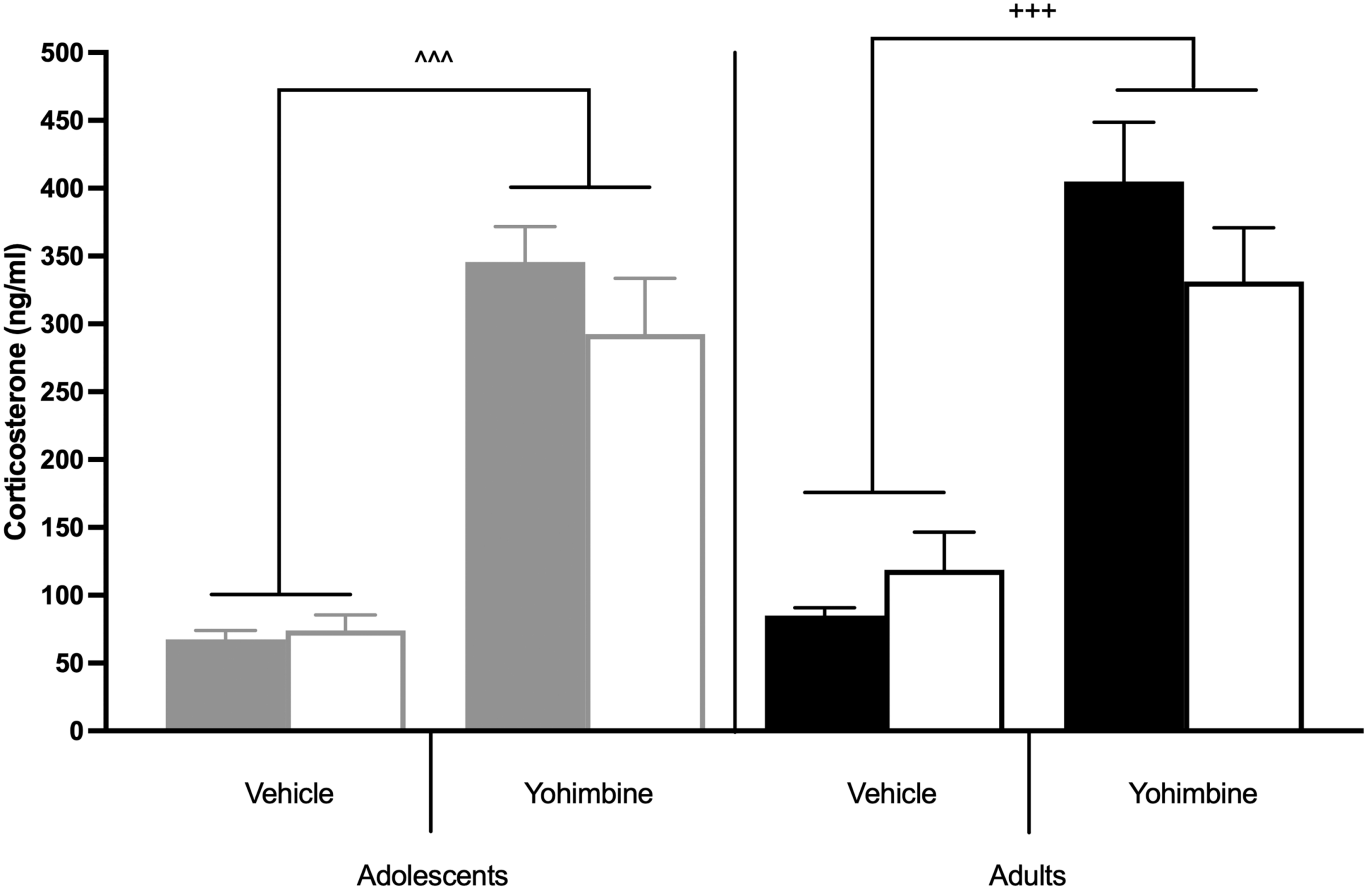
Yohimbine-induced HPA axis activation in behaviorally tested animals. The bars represent mean (±SEM) corticosterone levels after yohimbine or vehicle injections in adolescent (gray bars) and adult (black bars) rats that underwent stress-induced reinstatement of CSE- (solid) or nicotine-seeking (hashed). ^^^ *p* < 0.0001 vs. vehicle, adolescents; +++ *p* < 0.0001 vs. vehicle, adults. *n* = 4–6 per group.

Further analysis of yohimbine-induced corticosterone secretion was done at the end of the study to examine a possible role of HPA axis activation in stress-induced reinstatement (Figure 6). There were main effects of Treatment [*F*(1,27) = 57.927, *p* < 0.0001], but not of Age [F(1,27) = 0.559, *p* = 0.461] or Drug [F(1,27) = 0.556, *p* = 0.462], nor were there any interactions between Age, Drug, or Treatment [F(1,27) = 0, *p* = 0.993]. Yohimbine significantly enhanced corticosterone levels across all groups (*p* < 0.0001; Figure 6).

## 4. Discussion

There is evidence of an age effect in smoking cessation such that early cigarette use is associated with greater dependence and difficulty quitting (20, 21). Furthermore, early teen use of e-cigarette is associated with increased odds of cigarette smoking (34). Despite these clinical findings, there have been few studies that have examined reinstatement to nicotine-seeking behavior in adolescents (35, 36). In the present study, we aimed to determine whether tobacco smoke constituents increase stress-induced reinstatement of drug seeking behavior in adolescent male rats, as we have previously found in adults (26).

Yohimbine, an α2 adrenergic receptor antagonist, was used as a pharmacological stressor since this had been shown to produce more reliable yohimbine-induced reinstatement of drug-seeking behavior than foot-shock in adults (23, 24). A dose response curve for yohimbine was constructed to determine an optimum dose at which both adolescents and adults showed yohimbine-induced corticosterone release. We show that adolescents and adults display distinct dose–response patterns of yohimbine-induced corticosterone secretion, with adolescents showing a monotonic increase whereas adults show significant effects only at the lowest and highest dose. Such findings of age differences in stress responses are consistent with functional immaturity of the hypothalamus-pituitary–adrenal (HPA) axis of adolescents as compared to adults, as has been shown in other studies (37, 38). Since the highest yohimbine dose (2.5 mg/kg) examined induced significant corticosterone secretion in both adolescents and adults, this dose was chosen for subsequent analysis of stress-induced reinstatement. When yohimbine (2.5 mg/kg) was once again tested at the end of the behavioral study, no differences in stimulated corticosterone levels were found between the animals in the two age groups. It is not clear whether this reflects prior behavioral treatment and drug exposure, or whether it reflects that animals that started the experiment in early adolescence were now approaching adulthood. Although this might be considered an experimental confound, it should be noted that animals were exposed to different reinstatement conditions in a counterbalanced design to control for ongoing developmental changes.

### 4.1. Influence of tobacco constituents and age on self-administration, extinction, and reinstatement

Consistent with our earlier findings (27), there was no difference in self-administration of nicotine and CSE at an equivalent nicotine dose. However, age differences were evident with adolescents having higher reinforced and non-reinforced responding than adults. No differences in extinction of responding for CSE and nicotine were observed at each age, although some age differences were observed. Consistent with earlier findings (35), adults extinguished more slowly than adolescents at initial time points. However, in contrast to earlier findings, we found that adolescents extinguished more slowly at later stages. Despite this, all animals reached extinction criterion by day 6.

Whereas tobacco constituents did not impact self-administration or extinction, they had a significant effect on reinstatement behavior. Although previous studies have shown that cues can induce reinstatement of nicotine-seeking behavior in adult animals (26, 33, 39, 40), we now show that, regardless of age and drug, animals do not show significant cue-induced reinstatement of drug-seeking behavior as defined by difference from extinction baseline. The differences observed may be due to methodological differences in which other studies used multiple cues [tone and light; (39), substituted saline for nicotine during reinstatement (39), or only studied adults (26, 33)]. Alternatively, this may reflect the difference between social housing of animals, as done in the current study, as compared to individual housing done in prior studies (41). Studies have shown individually housed rats display an anxiogenic phenotype (42, 43) and show increased sensitivity to reward-related cues (41, 44). Although no groups showed significant cue-induced reinstatement as compared to extinction baseline, all animals that had previously self-administered CSE showed significantly higher cue-induced responding than those that had self-administered nicotine, regardless of age. We now also show that yohimbine + cues induce greater reinstatement in both adolescent and adult animals that previously worked for CSE as compared to those that worked for nicotine alone. This finding in is slightly different from that of our earlier work (26), in which CSE enhanced reinstatement in animals that received yohimbine without cues. This difference may reflect experimental variables, particularly single housing in the earlier study as compared to group housing in the current study. Our current results, however, are in line with those of Costello in that non-nicotine tobacco smoke constituents present in CSE enhance reinstatement of drug-seeking behavior. Our finding of lack of age differences in reinstatement of nicotine-seeking behavior is consistent with that of Shram et al. (35) who showed that nicotine priming induced similar reinstatement profiles in adolescent and adult rats.

Previous reports indicate a role of sex in the reinforcing effects of nicotine (45), while others have observed no sex differences in stress-induced reinstatement of nicotine-seeking in adult rats (33). However, sex differences in the effects of yohimbine to enhance the reinforcing efficacy of nicotine self-administration in adolescent animals have been reported, with females showing greater sensitivity (46). To date, however, there have been no studies comparing sex differences in the reinstatement of nicotine-seeking behavior in adolescent animals, or of the impact of tobacco smoke constituents. Given ample clinical data that females, both adolescent and adult, have greater difficulty quitting smoking (47, 48), such studies are required.

## 5. Conclusion

This study provides further experimental evidence indicating the importance of age and tobacco smoke constituents in animal models of tobacco dependence. Our laboratory has previously shown that age plays a greater role than the presence of non-nicotine tobacco smoke constituents in acquisition of drug self-administration (27). In the present study we confirm this and now show that age, but not tobacco constituents, impacts extinction. However, we find no age difference in stress + cue-induced reinstatement of drug-seeking behavior, but greater reinstated responding in animals that previously worked for CSE. Thus, age and tobacco constituents have differing influence on early and late stages of nicotine-seeking behavior.

## Data availability statement

The raw data supporting the conclusions of this article will be made available by the authors, without undue reservation.

## Ethics statement

The animal study was reviewed and approved by Institutional Animal Care and Use Committee of the University of California, Irvine.

## Author contributions

CG, JB, and FL were responsible for experimental concept and design. CG performed all experiments and wrote early drafts of the manuscript. CG and DC analyzed data and created figures. DC and FL provided revisions of the manuscript. JB and FL consulted on statistical analysis. All authors contributed to the article and approved the submitted version.

## Funding

Funding was provided by NIH grant DA 040440.

## Conflict of interest

The authors declare that the research was conducted in the absence of any commercial or financial relationships that could be construed as a potential conflict of interest.

## Publisher’s note

All claims expressed in this article are solely those of the authors and do not necessarily represent those of their affiliated organizations, or those of the publisher, the editors and the reviewers. Any product that may be evaluated in this article, or claim that may be made by its manufacturer, is not guaranteed or endorsed by the publisher.
